# Specific Learning Disability Unmasked by Psychophysiological Insomnia

**DOI:** 10.7759/cureus.7480

**Published:** 2020-03-30

**Authors:** Udayakumar Narasimhan, Fatima Shirly Anitha, Monika Battula

**Affiliations:** 1 Pediatrics, Sri Ramachandra Institute of Higher Education and Research, Chennai, IND

**Keywords:** dyslexia, insomnia, specific learning disability

## Abstract

Sleep is essential for good cognition and academic performance. Children with deficits in learning and cognition thus have various sleep disturbances. Learning disabilities in dyslexia are exaggerated with associated sleep problems which also impact the daily routine. Although it is essential to rule out organic etiologies in insomnia, it should also be borne in mind to probe the child's functional performance in school. Psychophysiological insomnia should be considered in perpetuating stressful conditions. Any scholastic difficulty in a child has to be seriously evaluated to estimate the underlying condition. We report a rare case of psychophysiological insomnia diagnosed in a child with specific learning disability. Awareness of the associated sleep difficulties in dyslexia helps in improved assessment and treatment of such children.

## Introduction

Sleep disorders are common complaints encountered in pediatric office practice with a prevalence of 3.7% globally [1]. The prevalence is significantly lower than that estimated in epidemiological studies, and this indicates underdiagnosis of sleep disorders by primary care providers. Insomnia in children could be due to organic etiologies or psychiatric illnesses, and hence a structured approach will unmask an array of these conditions. Learning and cognitive skills of children depend on good quality of sleep. Sleep disorders were found to have a higher prevalence in children with dyslexia [2]. We report a rare case of psychophysiological insomnia diagnosed in a nine-year-old boy, the evaluation of which unmasked his learning disability.

## Case presentation

A nine-year-old male presented to the pediatric outpatient department of our tertiary care hospital with complaints of sleep disturbances for the past four years and poor scholastic performance for the past two years. He had change of school two years back to a tougher curriculum. He was currently studying in fourth grade and was managed with various medications which offered no relief. There was no history of fever, seizures, altered sensorium or atopic conditions. He had no symptoms of gastrointestinal disturbances, head injury, substance abuse or chronic drug intake. Sexual abuse was also ruled out by detailed evaluation by a child psychiatrist. Detailed sleep history and review of sleep diary revealed his difficulty in falling asleep for nearly four to five days per month, characterized by being completely awake for two days even without short naps in between. This did not affect his routine in the subsequent days. He had difficulty in Mathematics and English in academics. He was the first sibling and the index case for high scholastic expectations from his parents. There was no history of inattention, hyperactivity, mood changes or history of being bullied by his friends. He was the first born of a third-degree consanguineous marriage, with a four-year-old younger brother. His perinatal period was uneventful with normal development. There were no conflicts, recent deaths or history of sleep problems in the family. His screen time was two hours per day, and both brothers shared the same room without television. There was no excessive intake of caffeine.

On examination, he was a cheerful co-operative child, moderately built and nourished with a weight of 26 kg and a height of 126 cm. There was no pallor, facial dysmorphism or neurocutaneous markers. His vitals, anthropometric measurements and systemic examination were within normal limits. His behavioral observation showed that his attention was easily aroused with sustained eye contact. He was able to comprehend and follow instructions adequately. Investigations such as serum hemoglobin, ferritin, vitamin B12, vitamin D levels, hepatic and renal parameters with electrolytes were normal. The MRI scan of the brain and the sleep electroencephalogram (EEG) showed increased cortical arousal during the sleep onset period.

This child was simultaneously evaluated in our child development unit for his difficulty in scholastic performance. Wechsler Intelligence Scale for children (WISC-4 India) was administered where he obtained an overall intelligence quotient (IQ) score of 88 (below average) with some scatter in the subscales [3]. Dyslexia Screening Tool Junior (Indian version) showed “at risk quotient” (ARQ) of 1.4, which indicates "strong risk." Sentence Completion Test Junior showed no significant conflicts in school adjustments. He found Mathematics difficult but had a positive attitude towards his future professional achievements. He was preoccupied with sleep problems and had fears related to darkness. No significant emotional themes were identified in Children’s Apperception Test (CAT). Controlled Projective Test (CPT) showed his prominent need for affiliation with peers to resolve conflicts and his potential fear of being rejected by peers. Conners Self-Report Scale showed that he did not report any specific concerns. Our patient was diagnosed to have chronic insomnia by the International Classification of Sleep Disorders (ICSD-3) criteria [4]. His symptom characteristics like exaggerated physiological arousal, learned sleep preventing measures, excessive worry regarding sleep and a heightened concern regarding daytime consequences fit in with psychophysiological type of insomnia [5].

The results were discussed with his parents who were advised to have realistic academic expectations. They were advised to avail remedial education services as his sleeplessness could be a manifestation of underlying academic stress. The child was advised sleep hygiene measures with a prescription of melatonin (3 mg) orally once a day for 10 days, to be taken an hour prior to sleep to regulate his sleep pattern. As child development follows a biopsychosocial trajectory, his symptoms were managed accordingly which benefited the child. On review at three months, his sleep schedule was regular and he was coping better with academics. Thus a multidisciplinary approach, ruling out all organic etiologies and a detailed probe into his academics helped in the diagnosis of psychophysiological insomnia with specific learning disability.

## Discussion

Sleep is a developmental process which begins in utero and depends on the dynamic interaction of biological, psychological and social factors. Insomnia in children is a major comorbid condition which is often unnoticed. Sleep disturbances affect the child’s scholastic performance due to sleepiness with difficulty to concentrate in school and also cause emotional stress to the parents. Although it is well known that insomnia can cause cognitive disturbances, studies show that dyslexia can also be present with insomnia as a prime clinical feature [6].

Deficiency of iron, vitamin B12 and vitamin D are identified as nutritional causes of insomnia [7]. Sleep disorders are common in major organ impairment as in chronic kidney or liver disease [8]. Hyperthyroidism is also an important hormonal cause of insomnia [9]. EEG detects epileptiform activity as various epileptic syndromes occur during sleep, thereby causing insomnia and affecting cognition. Insomnia is also the most common sleep wake disorder in patients with primary brain tumors [10].

Insomnia is common in developmental disorders like Rett syndrome, Williama syndrome and Angelman syndrome. It is commonly associated with autism, depression and other psychiatric conditions [11]. However, the association of dyslexia with insomnia is not much reported and less understood. A study by Carotenuto et al. showed an increased prevalence of sleep disorders in dyslexia [2]. This is attributed to the increase in number of sleep spindles and long periods of slow wave sleep which reflects on language learning [12]. Children with learning disability have significant deficits in learning new oral vocabulary, thus explaining the link between sleep disturbances and language difficulties [13].

Our patient with dyslexia would have managed his initial grades with subtle difficulty. However, with a higher grade syllabus and tougher curriculum his learning disability had become obvious and he found it difficult to cope up with the pressure. Psychophysiological insomnia is common in older children and adolescents [14]. The causes attributed include genetic predisposition, underlying medical or psychiatric conditions and perpetuating factors such as caffeine intake and poor sleep habits. The perpetuating factor here was the high parental expectations, and the precipitating factor being his learning disability which manifested as insomnia in this child with probable genetic predisposition. His parents sought medical attention primarily for his sleeplessness the evaluation of which revealed this underlying learning disability.

## Conclusions

Insomnia in children should be viewed as a cluster of symptoms and investigated extensively to identify the cause and triggers. Our case report stresses on the fact that poor scholastic performance in children should be seriously considered and investigated in detail even in the presence of obvious complaints. Psychophysiological insomnia, although rare, needs to be considered after ruling out organic etiologies. This case is reported for its rarity and for the association between insomnia and specific learning disability.
